# Checking the validity and reliability of the Japanese version of the Mini-Cog using a smartphone application

**DOI:** 10.1186/s13104-022-06101-4

**Published:** 2022-06-25

**Authors:** Yoshinobu Saito, Sho Nakamura, Ayumi Tanaka, Ryo Watanabe, Hiroto Narimatsu, Ung-il Chung

**Affiliations:** 1 Faculty of Sport Management, Nippon Sport Science University, 1221-1 Kamoshida-cho, Aoba-ku, Yokohama, Kanagawa 227-0033 Japan; 2 Center for Innovation Policy, Kanagawa University of Human Services, Research Gate Building TONOMACHI 2-A, 3-25-10 Tonomachi, Kawasaki-ku, Kawasaki-shi, Kanagawa, 210-0821 Japan; 3 Cancer Prevention and Control Division, Kanagawa Cancer Center Research Institute, 2-3-2 Nakao, Asahi-ku, Yokohama, Kanagawa 241-8515 Japan; 4 Graduate School of Health Innovation, Kanagawa University of Human Services, Research Gate Building TONOMACHI 2-A, 3-25-10 Tonomachi, Kawasaki-ku, Kawasaki-shi, Kanagawa, 210-0821 Japan; 5 Division of Health Promotion, Fujisawa City Health and Medical Foundation, 5527-1 Oba, Fujisawa, Kanagawa 251-0861 Japan; 6 Department of Bioengineering, Graduate School of Engineering, The University of Tokyo, 7‐3‐1 Hongo, Bunkyo‐ku, Tokyo, 113‐8655 Japan

**Keywords:** Cognitive function, Smart phone application, Mobile health, Public health

## Abstract

**Objective:**

Cognitive decline is an important and well-documented health problem. The Mini-Cog, a simple cognitive function test, is recommended as a potential early cognitive screening tool. Kanagawa Prefecture, one of the largest prefectures in Japan, developed this self-testing application on a smartphone to enable a large number of residents to assess their cognitive function. This study aimed to verify the validity and reliability of the Mini-Cog.

**Results:**

Twenty men and 20 women aged 65–85 years were enrolled. Criterion-related validity of the method tested by professional staff was found to have an acceptable positive association. The test–retest reliability was lower than the clinically expected intraclass correlation coefficient value because of the inclusion of learning and order effects. If the Mini-Cog score of this application is low, the system is equipped with a function that advises the users on preventing cognitive decline, directing them to the appropriate services, and recommending visits to a medical institution. Therefore, the system can be used continuously as a tool for health behaviors and promotions.

**Supplementary Information:**

The online version contains supplementary material available at 10.1186/s13104-022-06101-4.

## Introduction

Cognitive decline is an important and well-documented health problem. Identifying older adults with dementia is important for planning their care needs and treatments. Screening tests to identify dementia, including the Mini-Mental State Examination (MMSE) [1], Clinical Dementia Rating [2], Montreal Cognitive Assessment [3], and Mini-Cog [4, 5], are widely used. Among them, the Mini-Cog is the simplest screening test that consists of a three-word recall task and a clock drawing test. Therefore, it is useful when clinicians have limited time and resources to administer the test [6].

The Mini-Cog was originally developed in a study of ethnolinguistically diverse community-aged Americans to screen for dementia in primary care settings. This original version of the study could be administered in an average of 3.2 min and showed high sensitivity (99%) and specificity (96%) [4]. In a population-based post hoc examination with participants from a random sample of 1119 older adults, the Mini-Cog and the MMSE had similar sensitivity (76% vs. 79%) and specificity (89% vs. 88%) [5].

Recently, the Mini-Cog has been translated into various languages and is recommended as a potential early cognitive screening tool for memory impairment screening and general practitioner evaluation [7–9].

In March 2020, Kanagawa Prefecture, one of the biggest local governments in Japan developed the ME-BYO index, to measure the ME-BYO in the health management application, My ME-BYO Record [10]. ME-BYO is a concept that does not consider health and sickness as two separable conditions; it covers the entire process of continuous change in the physical and mental conditions between health and sickness [10]. The ME-BYO index visualizes an individuals’ current state of ME-BYO and future disease risk in numerical form and comprehensively quantifies the domains of lifestyle, physical function, cognitive function, and mental health and stress. The ME-BYO index was developed based on evidence and discussion among experts. The specific evaluation items included sex, age, body mass index, systolic blood pressure (lifestyle), the Mini-Cog (cognitive function) [4, 5], locomotive function [11], walking speed (physical function), and mind-monitoring system (mental health and stress) [12].

For the evaluation of cognitive function using the ME-BYO index, we adopted the Mini-Cog, which is a simple and recommended screening test. Therefore, we developed a smartphone application to enable residents to perform the test. The widespread use of an application that allows residents to measure their cognitive function easily in their daily lives could speed up the diagnostic process, improve accessibility, and lead to early prevention measures. Moreover, it could help improve the health of the individuals, their families, and the community. Although various applications for assessing cognitive function have been developed [13, 14], to our knowledge, no free application of the Mini-Cog has been validated and reliably self-administered by the residents. Therefore, this study aimed to verify the validity and reliability of the Mini-Cog measurement method used by the general public through an application on their smartphones.

## Main text

### Methods

#### Study participants

The participants were healthy older adults aged 65–85 years who attended a health promotion facility. The target number of participants was 40 (20 men and 20 women). In the sample size design, the required number of participants was 29, assuming a significance level, power, and effect size of 5%, 80%, and 0.5, respectively. The final number of research subjects was set at 40, assuming dropouts and multiple applicants signing up simultaneously, as well as the burden on the study participants.

The Research Ethics Review Committee of the Graduate School of Health Innovation, Kanagawa Prefectural University of Health and Welfare approved this study (Approval No. Hodai No. 30-011). The purpose and content of the study were explained to the study participants in advance, and written consent was obtained before the study was conducted.

#### Measurement items

##### Mini-Cog assessment by the examiner and smartphone application

The Mini-Cog assessment consisted of a three-word recall task and clock drawing test. In the word recall task, a score of 0–3 points was assigned for each correct recall of 0, 1, 2, or 3 words. The word recall was allowed in any order. In the clock-drawing test, the participants were scored as “normal” or “abnormal” based on the placement of the clock hands. The Mini-Cog test was scored positive (dementia) if the delayed recall was 0 out of 3, or if the delayed recall was 1 or 2, and the clock drawing test was abnormal. It scored negative (not dementia) if the delayed recall is 3 points or if the delayed recall is 1 or 2 points and the clock drawing is normal [4]. Mini-Cog has developed currently 21 language versions, including Japanese, which is available for download from the Mini-Cog website [15].

The basic procedure and scoring criteria for the Mini-Cog using the smartphone application were similar to those used by the examiner. In the initial three-word recall task, one of the six versions was played randomly over the audio, and the participants were instructed to memorize the three words. Next, the participants were instructed to drag and place numbers 1–12 in a circle representing a clock and move the hands of the clock to 11:10. The last three-word recall was performed using voice or text input (see Additional file 1: Fig. S1).

##### Mini-Cog evaluation using the application

The research participants assessed their Mini-Cog judgment using the ME-BYO index application on a research smartphone. Mini-Cog assessment was performed once, following voice instructions, and evaluated using a score (0–5 points) and judgments of normal and abnormal. Mini-Cog self-assessments by the study participants using the application were conducted at the beginning of the study and after 8 weeks to verify the test–retest reliability.

##### Mini-Cog evaluation by examiners

A public health nurse was trained in the Mini-Cog conducted assessment. To verify criterion-related validity, a similarly trained public health nurse conducted a face-to-face assessment 4 weeks after the self-assessment by the study participants, referring to a previous study [16]. The 3-word recall task was conducted using a different version of the first application of the ME-BYO index.

#### Statistical analysis

Mini-Cog scores and judgments (normal and abnormal) were used to assess criterion-related validity and test–retest reliability. For criterion-related validity, Spearman's rank correlation coefficient was used to evaluate the results of the self-administered application by the study participants at the beginning of the study and the results administered by the public health nurses after 4 weeks. The kappa statistic was used to evaluate judgment (normal or abnormal).

Test–retest reliability was assessed using intraclass correlation coefficients (ICCs) (1,1) and 95% confidence intervals for the results of the self-administered application by the study participants and the retesting after 8 weeks. Statistical analyses were performed using SPSS version 27 (IBM Corp., Armonk, NY, USA) software. The significance level was set at 5%.

### Results

The characteristics of the participants during their participation in the study and the Mini-Cog values measured using the application are shown in Table 1. This study included 40 participants (20 men and 20 women). The mean age (standard deviation) was 74.9 (± 5.2) years. The mean Mini-Cog score (standard deviation) was 4.2 (± 1.0) points. Thirty-eight participants (95.7%) had a normal Mini-Cog judgment (3–5 points) and two (5.0%) had an abnormal judgment (0–2 points).

**Table 1:** Characteristics of the study participants

	Men (n = 20)		Women (n = 20)		Total (n = 40)	
Age, years	75.3	(5.3)	74.6	(5.2)	74.9	(5.2)
Educational attainment, %						
< 13 years	8	(40.0)	10	(50.0)	18	(45.0)
≥ 13 years	12	(60.0)	9	(45.0)	21	(52.5)
Missing	0	(0)	1	(5.0)	1	(2.5)
Working status, %						
Working with income	6	(30.0)	5	(25.0)	11	(27.5)
Not working	14	(70.0)	14	(70.0)	28	(70.0)
Missing	0	(0)	1	(5.0)	1	(2.5)
Living arrangement, %						
With others	19	(95.0)	16	(80.0)	35	(87.5)
Alone	1	(5.0)	3	(15.0)	4	(10.0)
Missing	0	(0)	1	(5.0)	1	(2.5)
Body mass index, kg/m^2^	23.1	(1.7)	21.2	(2.1)	22.2	(2.1)
Systolic blood pressure, mmHg	138.4	(13.3)	131.8	(18.7)	135.1	(16.3)
Diastolic pressure, mmHg	75.7	(10.5)	71.4	(9.5)	73.5	(10.1)
Mini-Cog score, score	4.0	(1.3)	4.5	(0.6)	4.2	(1.0)
Mini-Cog judgment, %						
Normal	18	(90.0)	20	(100)	38	(95.0)
Abnormal	2	(10.0)	0	(0)	2	(5.0)

Mini-Cog judgment normal score: 3–5; mean (standard deviation); n (%)

The results of the validation and reliability of the Mini-Cog are shown in Table 2. The Spearman's rank correlation coefficient was ρ = 0.442 based on the criterion-related validation of the ME-BYO index application scores at the beginning of the study and after 4 weeks. This indicates a positive correlation and a 100% agreement rate with the Mini-Cog judgment.

**Table 2:** Validity and reliability of the Mini-Cog by study participants and medical staff

	Mini-Cog score	Agreement of two categories		
	*ρ*, ICC (95% CI)	N	%	ICC (95% CI)
Criterion-related validity	0.442	40	100	–
Test–retest reliability	0.381 (0.085–0.616)	37	92.5	0.362 (−0.201–0.924)

Criterion-related validity: Pearson’s correlation coefficient; test–retest reliability: ICC (1,1)

*ICC* intraclass correlation coefficient, *CI* confidence interval

The results of the test–retest reliability of the ME-BYO index application scores at the beginning of the study and the reassessment after 8 weeks showed that the ICC (95% confidence interval) was 0.381 (0.085–0.616) and the Mini-Cog judgment was 0.362 (− 0.201–0.924), indicating poor reliability.

### Discussion

In this study, we developed a Mini-Cog application that can be self-administered by residents using a smartphone and tested its criterion-related validity and test–retest reliability.

Criterion-related validity was evaluated using the self-administered Mini-Cog results at the beginning of the study and those administered by medical professionals after 4 weeks. A positive correlation (ρ = 0.44) was observed between the scores, and the agreement rate of the Mini-Cog judgment was 100%. The evaluation of this application depends on the proficiency level of the smartphone, especially in the clock drawing test. The correlation coefficient of a previous study that validated the Thai version of the Mini-Cog face-to-face survey with the MMSE was 0.47 [17], which is comparable to the results of this study. Therefore, the method of measuring the Mini-Cog using a smartphone application was acceptable.

When the individuals repeated the Mini-Cog using the ME-BYO index application after 8 weeks, the test–retest reliability was lower compared to the clinically expected ICC value (≥ 0.6). In this study, the same test was repeated three times over a certain period, similarly to previous studies. Therefore, the inclusion of learning and order effects may have affected the results [mean (standard deviation)] of the Mini-Cog scores at the time of participation in the study: in the beginning 4.2 (± 1.0); 4 weeks later: 4.8 (± 0.5); 8 weeks later: 4.6 (± 1.0) [18].

One issue with the original version of the Mini-Cog is that it overestimates the positive predictive value, especially for mild cognitive impairment [6]. However, it can be administered in a short period and is recommended as a convenient screening test in areas where time and resources are limited [7–9]. If the score of this application is low, the system is equipped with a function that provides advice on preventing cognitive decline, directing users to appropriate services, and recommending visits to a medical institution. Therefore, the system can be used continuously as a tool for health behaviors and promotions. It can also measure in a remote capacity during an infectious disease epidemic, such as coronavirus disease 2019. In the future, it is expected that the Mini-Cog application will be equipped with technology that will alert the user when the score starts to decrease. Moreover, only a few large-scale studies have been reported in the region using the Mini-Cog [6]. Utilizing the data from the Kanagawa Prefecture will contribute to the accumulation of evidence towards this issue.

To conclude, this application has sufficient scientific validity to test a large population. Therefore, it can be used as a tool to maintain and improve health behaviors.

## Limitations

This study did not use random sampling, and the participants were older adults attending health promotion facilities. Therefore, caution should be applied while applying the results to older adults with diverse backgrounds. Another limitation is that comparisons with other mobile applications were not conducted.

## Supplementary Information

**Additional file 1: Figure S1.** Screenshot of the Mini-Cog application.

## Data Availability

The datasets used and/or analyzed during the current study are available from the corresponding author upon reasonable request.

## Abbreviations

ICC: Intraclass correlation coefficient
CIs: Confidence intervals
