# Supplementary material for: Checking the validity and reliability of the Japanese version of the Mini-Cog using a smartphone application
Source: BMC Res Notes. 2022 Jun 25;15:222. doi: 10.1186/s13104-022-06101-4 (PMC9233764; doi:10.1186/s13104-022-06101-4)
Supplement: Supplementary file 1 — Additional file 1: Figure S1. Screenshot of the Mini-Cog application. [file 13104_2022_6101_MOESM1_ESM.docx]

Additional file 1


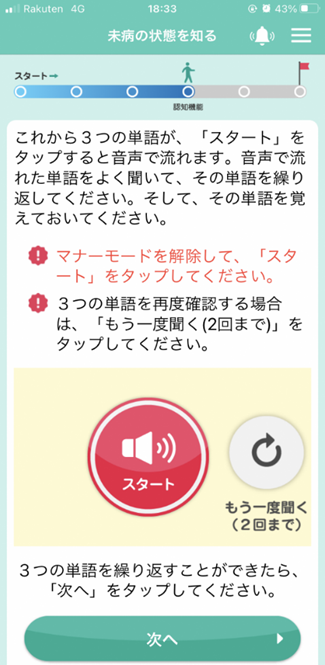

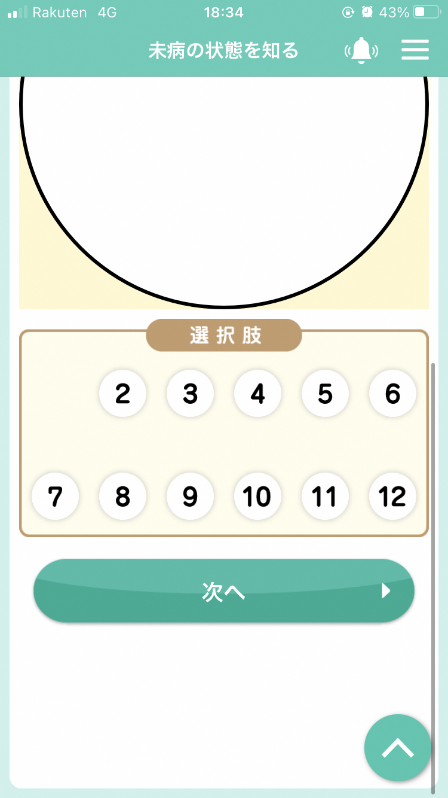

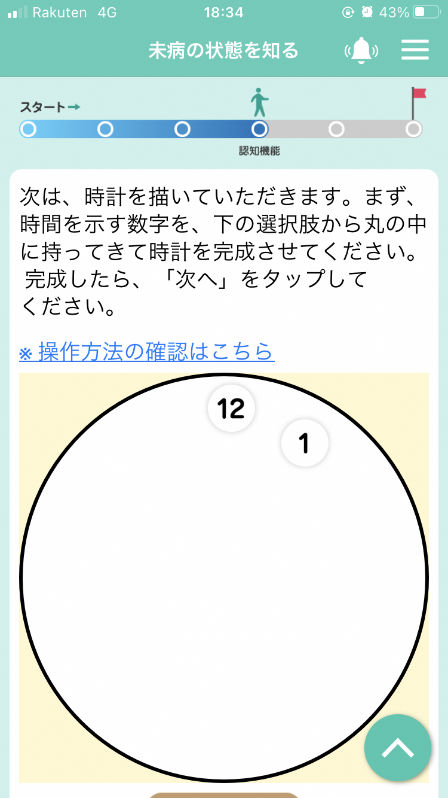

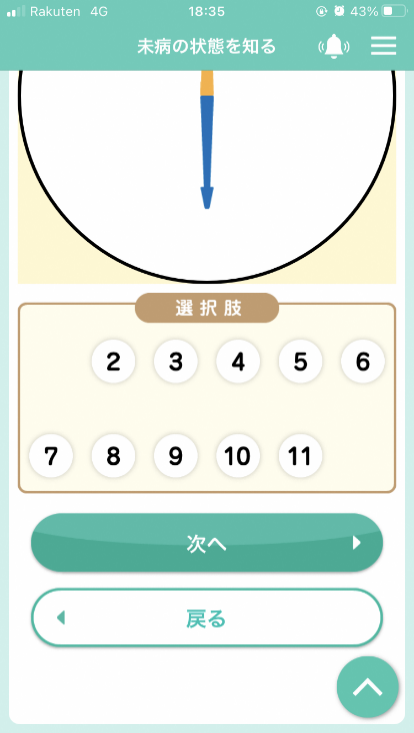

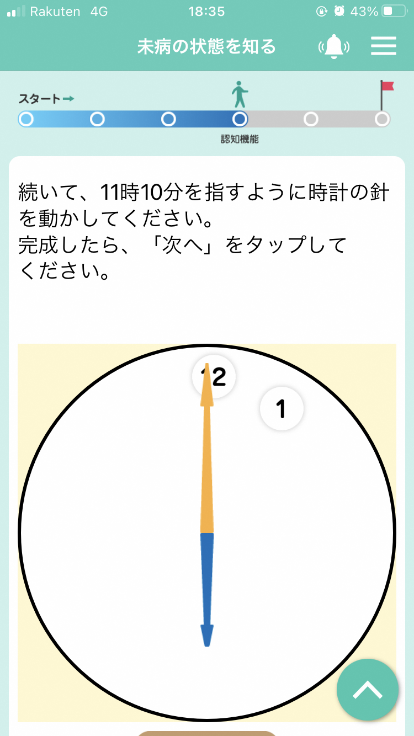

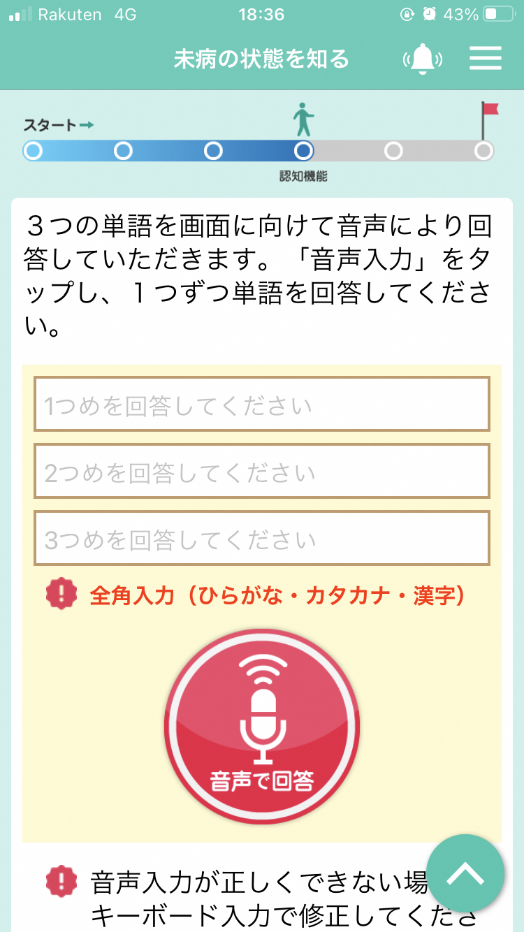

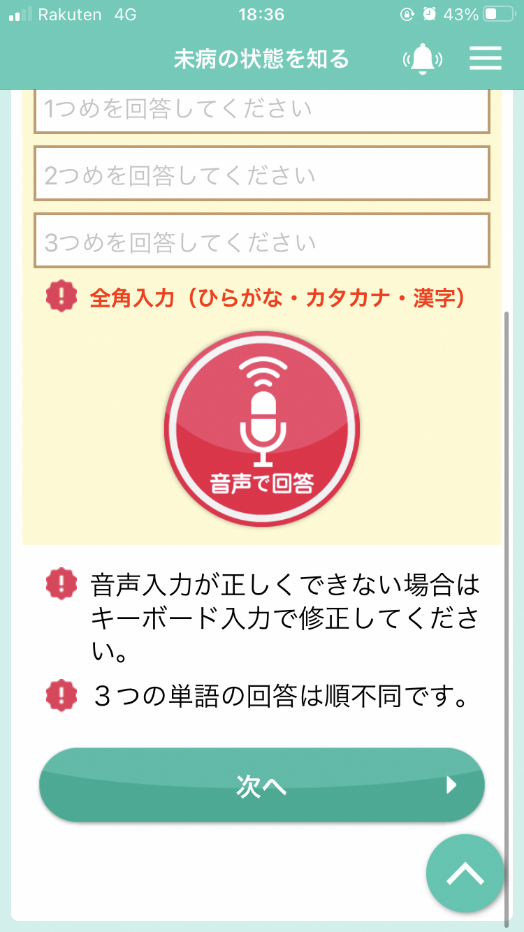


three-word recall task: memory

clock drawing test

three-word recall task: answer

Figure S1. Screenshot of the Mini-Cog application.
